# Exogenous application of NaBiF_4_ nanoparticle affects wheat root development

**DOI:** 10.1186/s12870-020-02348-w

**Published:** 2020-04-06

**Authors:** Yunfei Wu, Wangmenghan Peng, Zhaodi Dong, Qiuqing Jiang, Xurun Yu, Gang Chen, Fei Xiong

**Affiliations:** 1 Jiangsu Key Laboratory of Crop Genetics and Physiology, Yangzhou University, Yangzhou, 225009 China; 2 Co-Innovation Center for Modern Production Technology of Grain Crops, Yangzhou University, Yangzhou, 225009 China; 3 Joint International Research Laboratory of Agriculture & Agri-Product Safety, Yangzhou University, Yangzhou, 225009 China; 4 Jiangsu Key Laboratory of Crop Genomics and Molecular Breeding, Yangzhou University, Yangzhou, 225009 China; 5 Jiangsu Co-Innovation Center for Modern Production Technology of Grain Crops, Yangzhou University, Yangzhou, 225009 China

**Keywords:** Wheat, Root, Development, Nanoparticle, NaBiF_4_, Sodium, Homeostasis

## Abstract

**Background:**

Nanoparticle causes soil pollution, which affected plant development and then resulted in biomass decreased, especially in crops. However, little is known how sodium nanoparticles affect wheat root development at plant physiological level.

**Results:**

We used NaBiF_4_ (size of 50–100 nm) to analyze the effect in wheat development at plant physiological level. Under exogenous application of 50 μM NaBiF_4_ for treatment, wheat root elongation was inhibited, but fresh weight and dry weight were increased. We also found that NaBiF_4_ induced that the plant had lower content of sodium than negative control. Used no-sodium nanoparticle of BiF_3_ for another negative control, it was also supported that NaBiF_4_ entered into cell to replace of sodium and exported sodium out of plant. These results implied NaBiF_4_ might induce sodium export to maintain the balance between sodium and potassium elements. Additionally, metabolism analysis demonstrated that SOD activity was increased, but CAT and POD activity reduced under exogenous treatment of NaBiF4 nanoparticles.

**Conclusions:**

Sodium nanoparticles (NaBiF_4_) inhibited plant development by nanoparticle accumulation and sodium homeostasis broken, and then involved reactive oxygen species (ROS) signaling system response. These results provided more sights of sodium nanoparticle effect in plant development.

## Background

In the past several decades, the world’s population has been increased year by year. And cereal production similarly increased from 1.2 billion tons in 1969 to 2.8 billion tons in 2014 (FAOSTAT 1. data). Environmental factors play an essential role in crop plants development, such as temperature, light, drought, soil quality, nutrition, nanoparticles and so on. Environmental pollution, especially in soil, caused the crops production reduced due to affect root activity and impeded substance transport activity.

Many nanoparticles contribute to their promising suitability for solar cells, drug delivery, temperature sensors, indoor illumination, and field emission displays. Once nanoparticle is taken in through root pathway, it resulted in beneficial or opposite effect in plant development. Until now, several nanoparticles have been reported on the interactions with the plants, including carbonaceous nanomaterials (fullerenes and nanotubes), metal oxides, zero-valent metals, nanopolymers, QDs and other NPs (Ni(OH)_2_ and NaYF_4_) [1–8]. Actually, nanoparticles are different with nutrition, which are assimilated by root as anion or cation type. Base on the nanoparticle physical characteristic of composition, size, concentration and coating of nanoparticle, it plays different roles. To some degree, high concentrations or low concentrations of nanoparticles have opposite functions in plant development, as inhibited or promoted plants, respectively. Nevertheless, magnetic Fe_3_O_4_ even at the concentration of 2 mM does not cause serious injury in pumpkin (*Cucurbita maxima*) [9]. These positive effects of nanomaterials on plants were mainly reported for Au or Ag nanoparticles, Cu nanoparticles, Al related nanoparticles, TiO_2_ nanoparticles, CeO_2_ nanoparticles, SiO_2_ nanoparticles and carbonnanotubes [10–15].

Always, most of high concentrations of the nanoparticles caused phytotoxicity by toxic ions, cell or tissue damage, production of excess ROS, catalytic reactions [16–20]. To detect nanoparticles in the plant tissues, there are several different detection mechanisms of nanoparticles, such as fluorescence signaling, QDs, in situ analysis, nanoparticles color and so on [21]. Until now, little is known how nanoparticle affects crop plant development at metabolism level.

Wheat (*Triticum aestivum* L.) is one of the most important crop plants in the world, which supports the 1/3 of the food for human. Previously, it was reported that TiO_2_ nanoparticles with diameters ranging from 14 nm to 655 nm, were accumulated in wheat root. And TiO_2_ nanoparticles did not affect wheat seed germination, biomass and transpiration [22]. As the nanoparticles enter into plant cell, there are several different pathways for transport, such as: vascular system, membrane system, plasmodesmata system and so on. Base on the size of pathway in vascular, membrane, plasmodesmata or other system, we found nanoparticle size from 50 to 100 nm only depended on membrane. Previously, we used NaBiF_4_ and BiF_3_ for analysis the roles in rice root development [23, 24]. We found that NaBiF_4_ inhibited rice root elongation, but promoted more crown root formation. We analyzed several ROS signaling genes, which displayed transcript level of *OsOVP1*, *OsNIP2:1*, and *OsMT2* was reduced, but expression of *OsMT2b* increased [24]. Exogenous application of nanoparticle of BiF_3_for treatment, which did not reduce rice root elongation, but not mediate *OsOVP1*, *OsNIP2:1*, *OsMT2*, and *OsMT2b* transcript level changed [23]. Because the composition of these two nanoparticles, only one element (sodium) shows difference, which might interrupt the native balance system, for example, homeostasis of sodium-potassium balance.

Plants generally maintain a stable K^+^/Na^+^ ratio and a negative electrical membrane potential difference across the plasma membrane under a normal physiological state. Na^+^ enters into the roots through different channels and transporters [25]. However, if the balance was broken, plant may start ROS response reactions. In this study, we found that wheat root was much more sensitive to NaBiF_4_ nanoparticles than BiF_3_ nanoparticles in root development, which caused the balance of sodium potassium pump affected.

## Results

### Effect of nanoparticles on the wheat root development

**Fig. 1:**
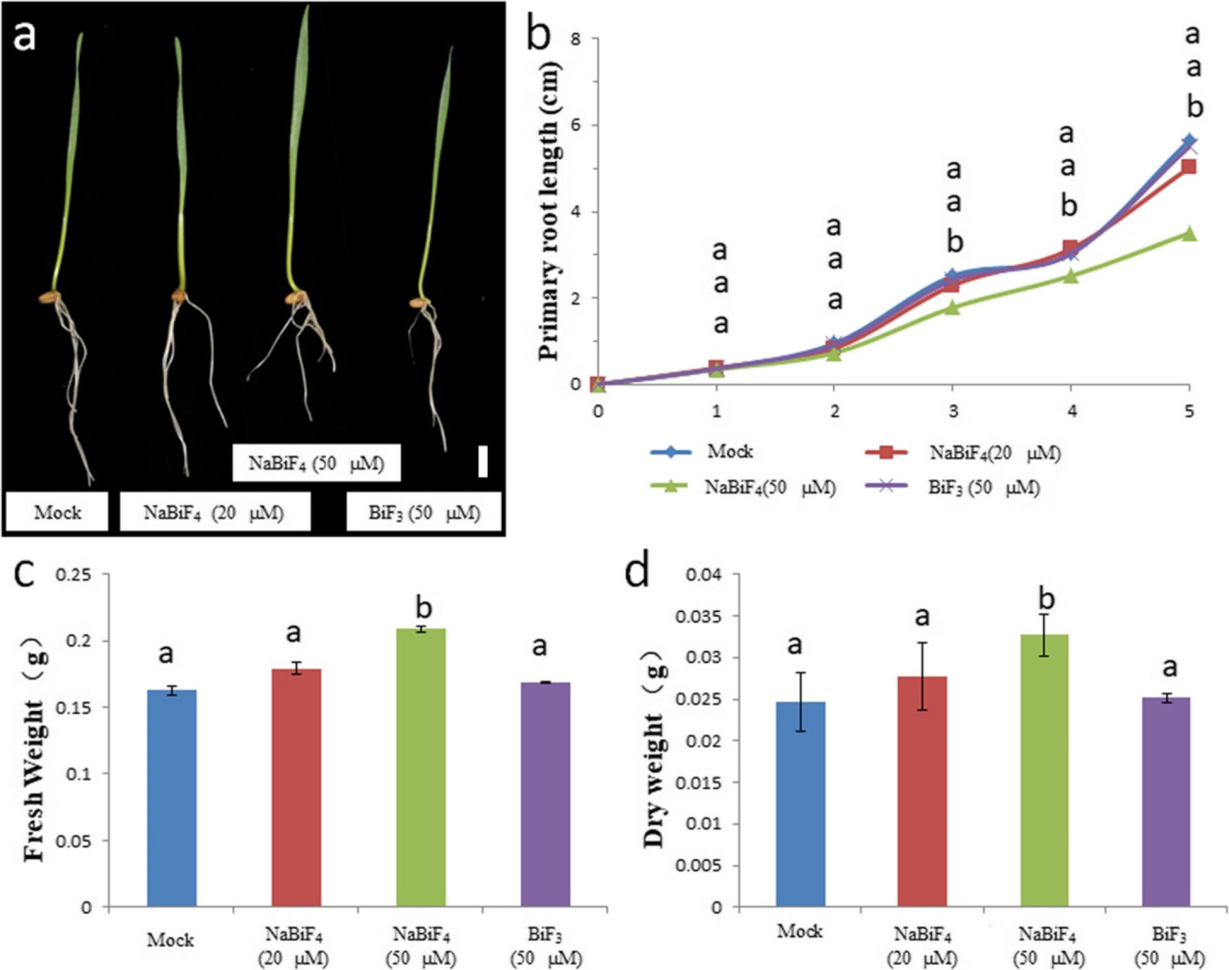
Effect of NaBiF_4_ nanoparticle in wheat development at seedling stage. **a** Images of wheat plants grown in MS0 medium with various concentrations of NaBiF_4_ nanoparticles at 10 DAG. Bar = 1 cm. **b** Primary root length as a function of DAG. **c** Fresh weight treated with different NaBiF_4_ nanoparticles at 10 DAG. **d** Dry weight treated with different NaBiF_4_ nanoparticles at 10 DAG. Error bars represent standard error for at least 5 samples. Values in the same column with different letters are significantly different (*P* < 0.05)

To analyze the effect of synthesized nanoparticles in wheat root development, wild type (WT) (*Triticumaestivum L* cultivar *Yangmai 13*) were grown in MS medium without sucrose (MS0), but with multiple concentrations of NaBiF_4_ nanoparticles. The images of the cultivated wheat were shown at 10 days after germination (DAG) in Fig. 1a. As demonstrated, the development of wheat root was significantly reduced by the 50 μM concentration of nanoparticles. Clearly, compared with that of the wheat grown on MS0 medium without nanoparticles as a negative control (Mock), the elongation speed of primary roots was much slower for the seedlings treated with 50 μM concentration of NaBiF_4_ nanoparticles (WT-HT) (Fig. 1b). And the length of WT-HT root reduced about 57.14%. Nevertheless, when the concentration of nanoparticles was declined to as low as 20 μM (WT-LT), the length of the primary roots was not significantly changed compared with the Mock (Fig. 1a-b). When the seedlings plants were treated with high concentration of NaBiF_4_ nanoparticles, the fresh weight and dry weight were measured. Interestingly, although the primary root elongation was inhibited, the fresh weight and dry weight were increased up to 131.25 and 130%, respectively (Fig. 1c-d). Here, we also used BiF_3_ nanoparticles as another controls, these data indicated that 50 μM NaBiF_4_ nanoparticles induced wheat biomass accumulation.

### Nanoparticles caused sodium export from wheat seedling plant

**Fig. 2:**
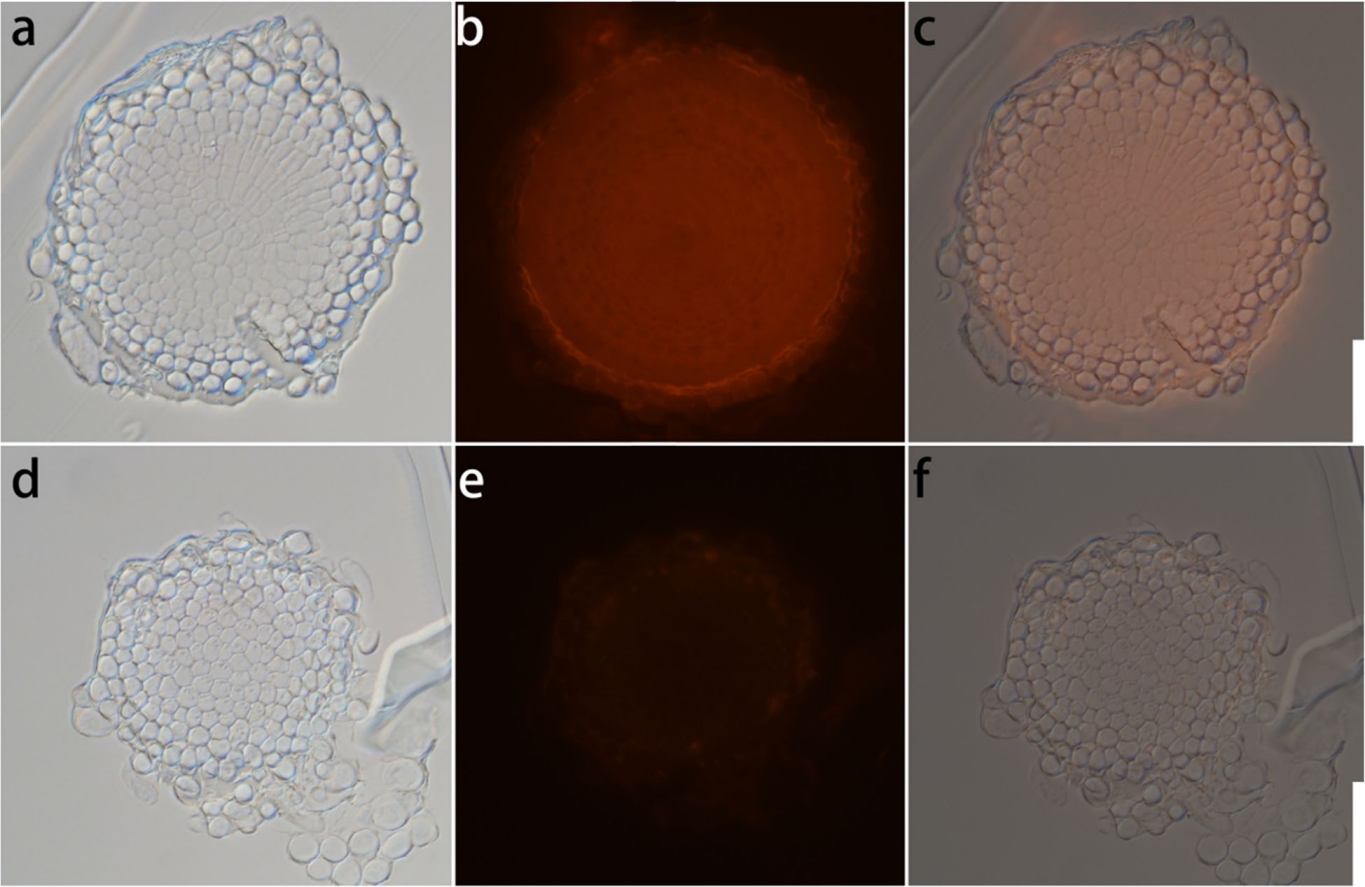
Cross section images of the wheat root tip treated with50 μM NaBiF_4_:Eu^3+^ nanoparticles at 4 DAG: **a** Bright channel, **b** RFP channel and **c** merged channel. Cross section images of the wheat root tip cultivated in MS0 medium without nanoparticles at 4 DAG: **d** Bright channel, **e** RFP channel and **f** merged channel. Bar = 50 μm

Previously, we reported that NaBiF_4_ nanoparticles caused rice root elongation inhibited due to phytotoxicity [24]. Eu acts as one type of earth element, which was visualized as red emission in the RFP channel. And, the NaBiF_4_:Eu^3+^ nanoparticles not only emitted dazzling visible red emission under the NUV excitation but also exhibited similar characteristic as the NaBiF_4_ nanoparticles in rice [24]. To get deep insight into the location of the nanoparticles, the cross section of root tip further confirmed that the NaBiF_4_:Eu^3+^ nanoparticles were distributed in the cells (Fig. 2a-c). Similarly, the negative results did not have any obvious signals in the wheat root grown in the MS0 medium (Fig. 2d-f). These results demonstrated that nanoparticles were accumulated in root tip. These results were similar with in rice, as the previous reported (Du et al., 2018).

**Fig. 3:**
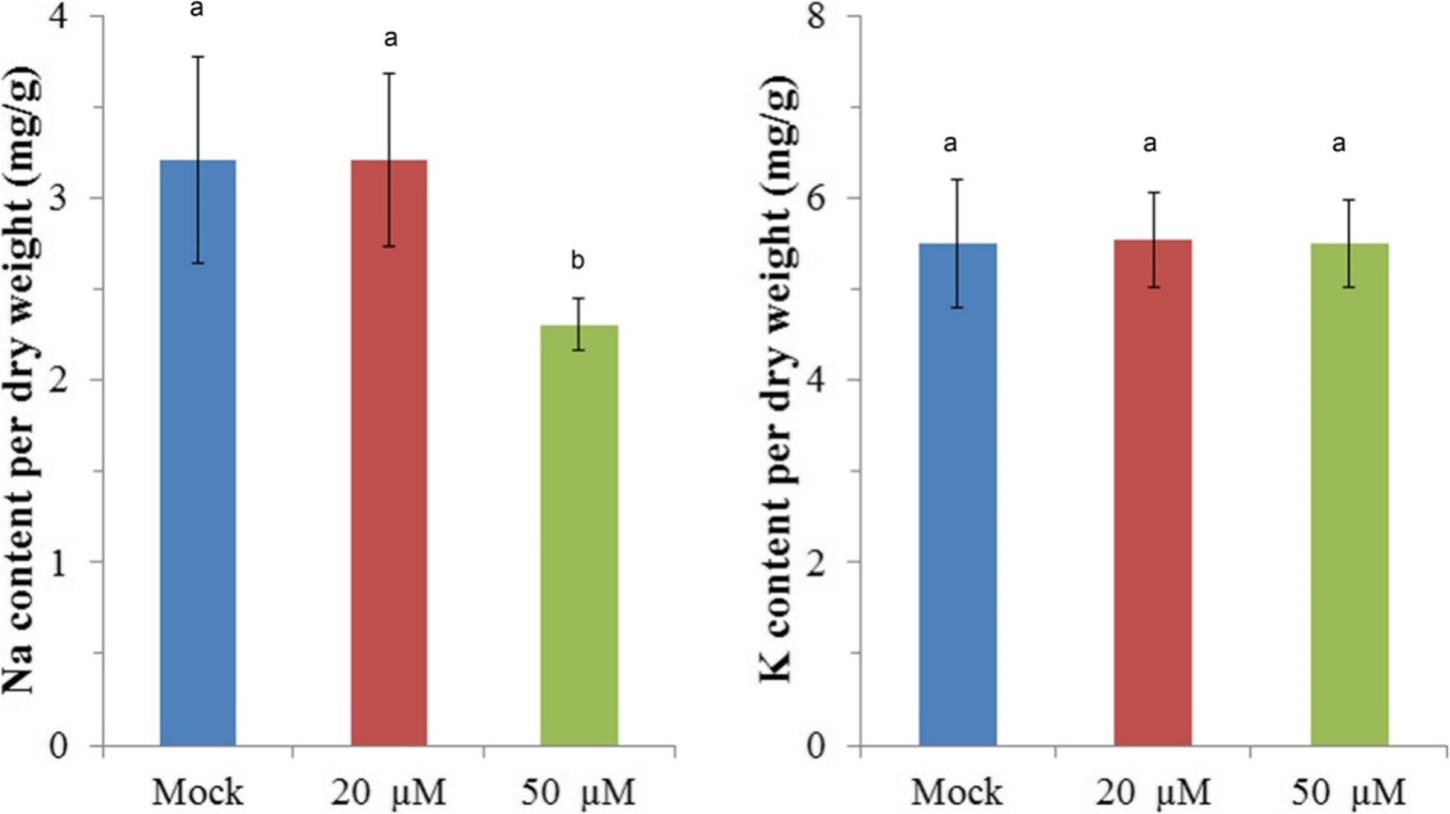
Sodium and potassium concentration of NaBiF_4_ nanoparticle treatment in wheat development. **a** Sodium concentration under different NaBiF_4_ nanoparticles treatment. **b** Potassium concentration under different NaBiF_4_ nanoparticles treatment. Error bars represent standard error for at least 5 samples. 20 μM NaBiF_4_ (20 μM).50 μM NaBiF_4_ (50 μM). Values in the same column with different letters are significantly different (*P* < 0.05)

Multiple factors affect ROS signaling response by phytotoxicity, such as sodium stress, nutrition transport disrupt, and so on. To further understand the mechanism by nanoparticles treatment, we measured sodium concentration. We found wheat seedling by 50 μM NaBiF_4_ nanoparticles treatment had lower level of sodium (71.874%) than Mock, but 20 μM NaBiF_4_ nanoparticles treatment was not significant changed (Fig. 3a). Here, we used potassium content for negative control, which demonstrated that there were no obvious changed (Fig. 3b) in these three groups. It implied that NaBiF_4_ nanoparticles entered into cell resulted in less sodium in cell. Meanwhile, NaBiF_4_ nanoparticles induced sodium export from cell.

**Fig. 4:**
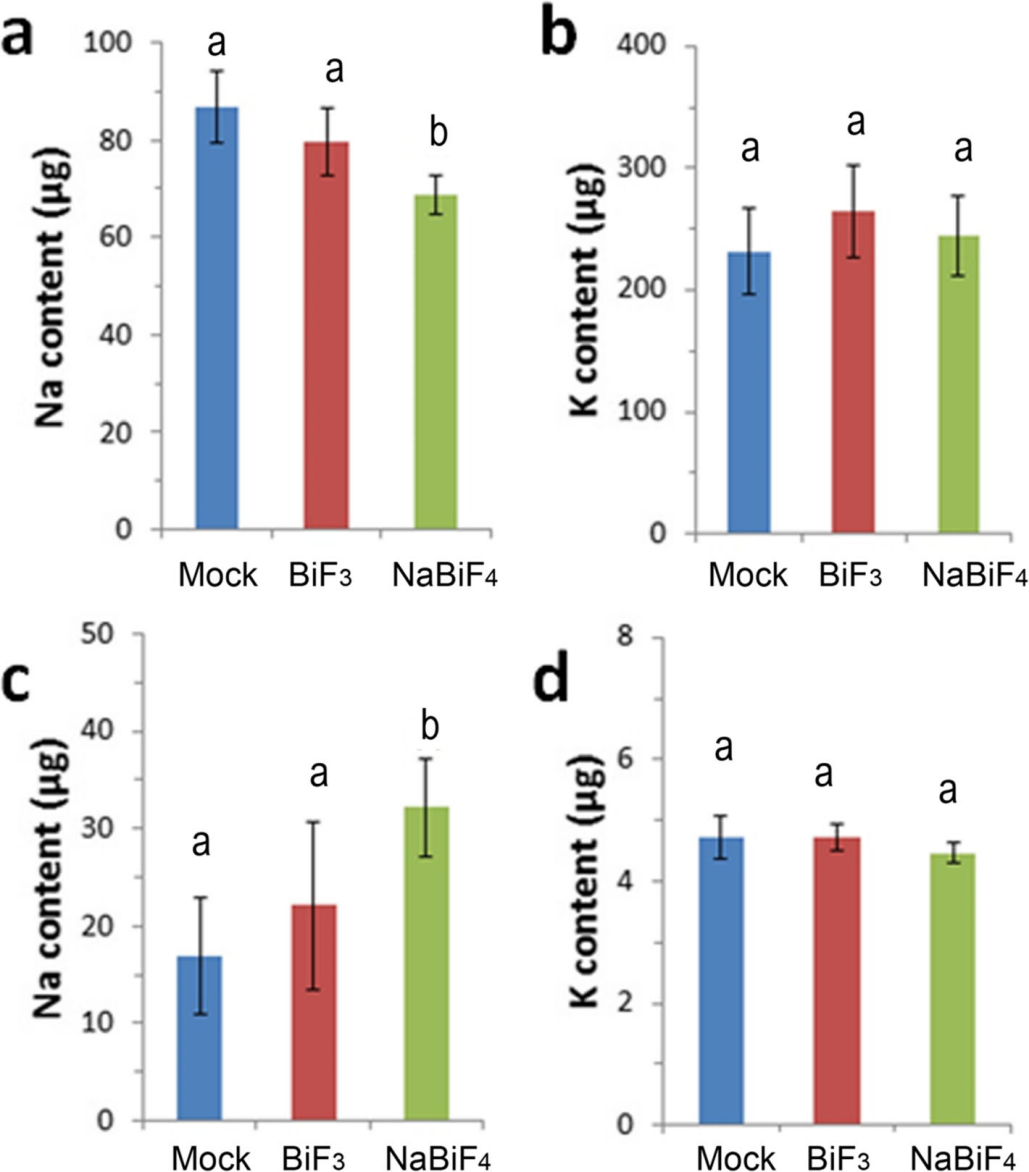
Sodium and Potassium concentration of NaBiF_4_ and BiF_3_ nanoparticle treatment in wheat development. **a** Sodium concentration under different NaBiF_4_ nanoparticles treatment in wheat. **b** Potassium concentration under different NaBiF_4_ nanoparticles treatment wheat. **c** Sodium concentration under different NaBiF_4_ nanoparticles treatment export from cell into water. **d** Potassium concentration under different NaBiF_4_ nanoparticles treatment export from cell into water. Error bars represent standard error for at least 5 samples. Values in the same column with different letters are significantly different (*P* < 0.05)

To further confirm this hypothesis, we used 50 μM NaBiF_4_ nanoparticles treatment for the similar experiments. And the solution used water to instead of MS0 medium in case sodium contamination from MS0 medium. With the treatment of NaBiF_4_ nanoparticles, sodium concentration was decreased about 81.39% compare with negative control. Also, we measured the sodium content in left solutions, sodium under NaBiF_4_ nanoparticles treatment had more than WT-CK (137.5%). And we also measured potassium concentration that there was no affected in Fig. 4b-d. This stated clearly that NaBiF_4_ nanoparticle caused extra sodium export out of plant into solution.

Additional, this phenotype might due to Bismuth (Bi) or Fluorine (F). we chose another nanoparticle BiF_3_ for synchronization. Exogenous application of 50 μM nanoparticle BiF_3_, which does not have sodium, did not inhibit root elongation in rice (Du et al., 2018a), as well as in wheat (Fig. 1). Also, with 50 μM BiF_3_ nanoparticles for treatment, sodium and potassium concentrations in plant were not affected in plant and export solutions (Fig. 4a-b). It further demonstrated that NaBiF_4_ displaced the sodium in cell to maintain the balance of sodium and potassium.

### ROS metabolism due to nanoparticles

**Fig. 5:**
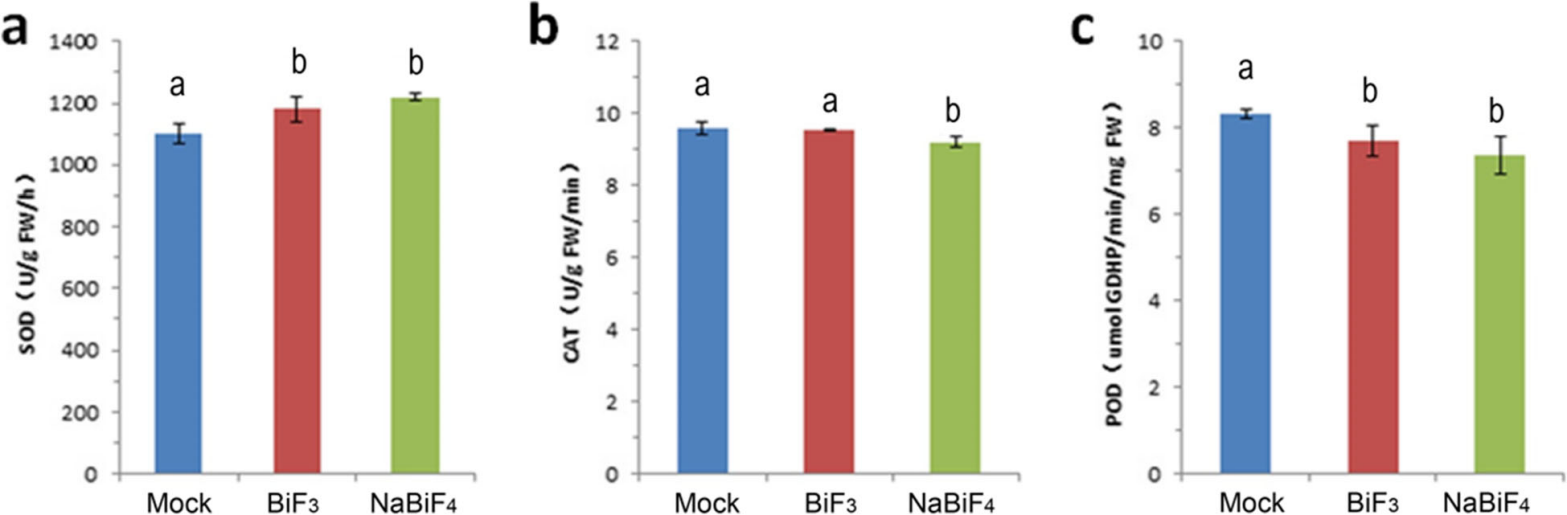
SOD, CAT, and POD Activity under NaBiF_4_ and BiF_3_ nanoparticle treatment in wheat development. **a** SOD activity level; **b** CAT activity level; **c** POD activity level; Error bars represent standard error for at least 5 samples. Values in the same column with different letters are significantly different (*P* < 0.05)

As deduced above, less sodium and much NaBiF_4_ nanoparticles entered into plant cells, which might affect cell metabolism reaction (phytotoxicity). This reaction includes two parts: affect sodium content, and xenobiotic substance, which induced by the nanoparticles might be the main factor to affect the development of the wheat roots. To response the phytotoxicity, several ROS system metabolism could be response to the wheat root, such as the superoxide dismutase (SOD), catalase (CAT), and peroxidase (POD) [11]. To better comprehend the nanoparticles induced phytotoxicity in the wheat root, the activity level of SOD, CAT, POD to several phytotoxicity related metabolism were analyzed in Fig. 5a-c. Compared with Mock, the activity of the SOD was much higher in these wheat roots treated with the NaBiF_4_ nanoparticles (50 μM), as well as treated with the BiF_3_ nanoparticles (50 μM). Noted that, with the treatment of the resultant nanoparticles, the activity level of the CAT and POD were reduced (Fig. 5b-c). Since the nanoparticles treated to the seedlings exhibited higher activity of SOD, and then lower activity of CAT and POD involved, it were expected to response to ROS system.

## Discussion

**Fig. 6:**
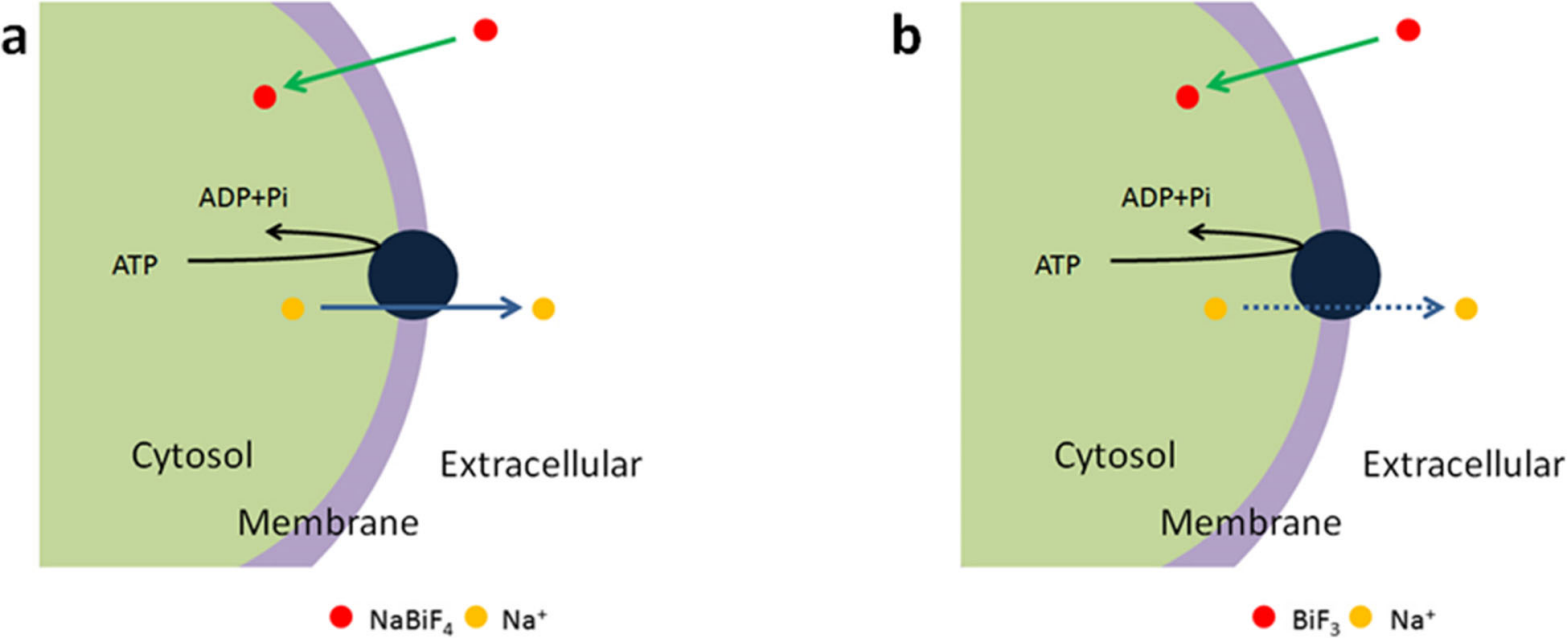
Model of reaction under NaBiF_4_ and BiF_3_ nanoparticle treatment in wheat development. **a**-**b** As NaBiF_4_ enters, sodium exported out of the cell; but BiF_3_ did not cause this phenotype

As the industry development, soil contaminated day by day due to heavy metal, salinization, nanoparticles accumulation. Previously, we used multiple concentrations of NaBiF_4_ and BiF_3_ for exogenous application to another crop plant (rice) for treatment. These results demonstrated that high content (100 μM) of NaBiF_4_ caused toxicity by the root length reduced and more crown root number. For the particles location, it is accumulated at division and elongation zone. Further phytotoxicity related genes, transcript level of *OsOVP1*, *OsNIP2;1*, and *OsMT2* was reduced and *OsMT2b* increased [24]. Similar content ofBiF_3_ exogenous treatment with NaBiF_4_ to rice did not show any obvious phenotype, although BiF_3_ also located at root tip, as NaBiF_4_ [23]. It implied that NaBiF_4_ and BiF_3_ have significant and different roles in plant development. In this study, we reported that same unsoluble nanoparticles, NaBiF_4_ and BiF_3_, which affected wheat development similar with in rice. Exogenous application of 50 μM NaBiF_4_ caused root length decreased, but BiF_3_ not. Interestingly, higher activity of SOD and lower of POD by the treatments of NaBiF_4_ and BiF_3_ nanoparticles, reduced CAT activity by NaBiF_4_, which demonstrated that both NaBiF_4_ and BiF_3_ affected ROS response reaction by tissue or cell abnormal in wheat root. Previously, Wang reported that nanoparticles caused phytotoxicity might due to (i) the dissolution and release of toxic ions; (ii) size- or shape-dependent mechanical damage and clogging; (iii) the production of excess ROS; (iv) binding interactions caused surface reconstruction of biological molecular structures; (v) oxidation of biomolecules through catalytic reactions [21]. Compare with BiF_3_ nanoparticles, NaBiF_4_ has one more element of sodium. We found less sodium concentration in plant than control, as well as used BiF_3_ treatment for negative control. Meanwhile, the reduced the sodium exported from the tissue into the solutions. It means that NaBiF_4_ play as sodium might cause sodium and potassium balance, BiF_3_ acts as one type of the exogenous substance, which might due to tissue damage and pathway clogging [21]. These results, above, indicated that NaBiF_4_ nanoparticles resulted in wheat root toxicity both in NaBiF_4_ accumulation in root and sodium export out of plant, as depicted as Fig. 6a. And BiF_3_ nanoparticles can also induce ROS signaling response only in BiF_3_ accumulation in root (Fig. 6b).

## Conclusion

Previously, we found that NaBiF_4_ accumulated at rice root elongation zone, and then induced ROS system signaling response by several genes transcript level affected, such as, *OsOVP1*,*OsNIP2;1*,*OsMT2*, and *OsMT2b*. Here, we used another crop plant, wheat, to further analyze these phytotoxicity reactions from plant physiological level. As the root assimilated NaBiF_4_ nanoparticle into cell, stable sodium from nanoparticle caused sodium export from root cell and then move into growth solution. Due to nanoparticle accumulation and less floating sodium level for plant physiological reaction, ROS related metabolism reactions were induced, which generated higher activity of SOD, and then lower activity of CAT, and POD. In the future, we will further analyze how nanoparticles move into cell.

## Methods

### Plant materials

The wheat cultivars selected in this study was Wheat (*Triticum aestivum* L. ‘*Ningmai13’*), which were provided by the Lixiahe Agricultural Research Institute.

### Synthesis of NaBiF_4_ and BiF_3_ nanoparticles

High-purity powders of NaNO_3_, Bi(NO_3_)_3_·5H_2_O, and NH_4_F acted as the raw materials to prepare the nanoparticles [23]. To prepare the NaBiF_4_ nanoparticles, two solutions were prepared. BiF_3_, NaBiF_4_ BiF_3_:Eu^3+^ and NaBiF_4_:Eu^3+^ were synthesized previous reported [23, 24].

### Determination of K^+^ and Na^+^ concentrations

The K^+^ and Na^+^ concentrations were measured as described previously [26–28].

### SOD, CAT, POD assay

The activities of SOD, CAT, and POD activity of wheat root was measured as described previously [29, 30].4 day after germination, the seedling wheat plants were move to 50 μM NaBiF_4_and BiF_3_ nanoparticles water solution for 3 days. About 100 mg of mixed material were harvested and ground in liquid nitrogen to a fine powder and then homogenized in 5 ml 10 mM PBS (pH 7.0) containing 1% PVP (w/v), 1 mM PMSF, 0.1% Triton-X100 (w/v) and 0.1 mM EDTA. The extraction was performed at 4 °C. After centrifugation at 12,000 g for 20 min, the supernatant solution was used as the preparation for individual enzyme activity. Then SOD and CAT activity were measured by spectrophotometer at 560 nm and 240 nm, respectively. The adrenochrome formation in the next 3 min was recorded at 470 nm in a UV–V is spectrophotometer.

### Statistical analysis

The experimental data was performed using t-test at a probability significance level of *P* < 0.05 in SPSS.

## Data Availability

All data generated or analyzed during this study are included in this manuscript.

## Abbreviations

ROS: Reactive oxygen species
SOD: Superoxide dismutase
CAT: Catalase
POD: Peroxidase
OsMT1: Metallothionein 1
OsMT2: Metallothionein 2
OsOVP1: Vacuolar H^+^-translocating inorganic pyrophosphatase
OsNIP2: 1: nodulin 26-like intrinsic protein
OsMT2b: Metallothionein2b
